# Editorial: Purple Haze: Issues on Cannabis Legalization

**DOI:** 10.3389/fpsyt.2021.796032

**Published:** 2021-11-10

**Authors:** Stéphane Potvin, Yasser Khazaal, Amine Benyamina, Marc N. Potenza

**Affiliations:** ^1^Centre de Recherche de l'Institut Universitaire en Santé Mentale de Montréal, Montreal, QC, Canada; ^2^Department of Psychiatry and Addiction, University of Montreal, Montreal, QC, Canada; ^3^Addiction Medicine, Department of Psychiatry, Lausanne University Hospitals, Lausanne, Switzerland; ^4^Unité Psychiatrie-Comorbidités-Addictions-Unité de Recherche PSYCOMADD 4872, Université Paris-Sud-APHP, Université Paris Saclay, Villejuif, France; ^5^Connecticut Mental Health Center, New Haven, CT, United States; ^6^Connecticut Council on Problem Gambling, Wethersfield, CT, United States; ^7^Wu Tsai Institute, Yale University, New Haven, CT, United States; ^8^Departments of Psychiatry, Child Study and Neuroscience, Yale School of Medicine, New Haven, CT, United States

**Keywords:** cannabis, legalization, cognition, mental health, driving

Considering the progressive legalization of cannabis across jurisdictions, we prepared a special topic that addresses significant issues relevant for future legalization initiatives. This topic seeks to: (i) characterize the personal characteristics of individuals who support recreational and medical cannabis legalization; (ii) characterize the profiles of people who use cannabis and related compounds such as tetrahydrocannabinol (THC) and cannabidiol (CBD); (iii) document the psychiatric and cognitive consequences of cannabis products, used either for recreational or medical purposes; and (iii) define priority areas deserving more research.

Using data from the 2016 *National Drug Strategy Household Survey* completed by 21,729 participants in Australia, Chiu et al. investigated the relationship between personal characteristics and support for cannabis legalization. Forty percent and 77% of participants supported the legalization of recreational and medical cannabis use, respectively. Cannabis use and high-risk drinking were associated with increased support of recreational and medical cannabis legalization. Nicotine use was only associated with increased support of recreational cannabis legalization. Although younger age was associated with greater support for legalization of recreational cannabis use, there was more support for legalization of medical cannabis use in older individuals. Psychological distress was associated with a higher likelihood of supporting recreational cannabis legalization, whereas support for medical cannabis legalization was stronger amongst individuals with chronic pain. Nevertheless, cannabis-use status was the strongest statistical predictor of support for both recreational and medical cannabis legalization.

People who use cannabis and related products for recreational and medical purposes do not form a homogeneous group of individuals, raising the need to characterize user profiles. Using data from an online survey completed by 329 people with “regular” use of cannabis, Amiet et al. examined the relationship between cannabis-use motives, expectancies, and profiles and psychological symptoms. Latent class analyses revealed two groups: those endorsing multiple motivations (social, coping, etc.) and higher positive and negative expectations of cannabis use, and those with low motives and expectancies. Individuals with High Motives and High Expectancies were more likely to meet criteria for cannabis use disorder (CUD) and report higher levels of anxious, depressive, and psychotic symptoms. These results are consistent with previous studies, thus defining modifiable targets (e.g., motives and expectancies) for future psychological interventions for CUD. Leveraging data from an online survey completed by 533 people who use cannabis and drink alcohol, Karoly et al. categorized participants into those who use cannabis for medical vs. recreational purposes. They determined that the former subgroup reported drinking less frequently than the latter group. In secondary analyses, they found that the use of high-THC/CBD was associated with more drinks on cannabis-use days. Such results demonstrate that cannabis and alcohol co-use is influenced by the reasons for cannabis use and cannabis content. On a related topic, Vilches et al. examined the potential differences between people who use CBD with and without cannabis co-use. Based on a survey completed by 182 respondents who reported using CBD, the authors noted that those with cannabis co-use were younger, had lower educational attainment, were more likely to use nicotine and to misuse alcohol, used more varied methods of CBD consumption (e.g., vaping, smoking, edible), and were more likely to report medical ailments such as sleep disorders. The association between cannabis and other substance use is consistent with previous studies.

The association between cannabis use and psychotic symptoms has been one of the most rigorously examined (1, 2). For instance, for those with a diagnosed psychotic disorder, there is reliable evidence showing that cannabis use is associated with poorer prognoses (3). Given that the psychotomimetic effects of cannabis are attributed to THC, and that the pharmacological effects of THC can be (partially) reversed by CBD in rodents (4), it has been hypothesized that CBD may be considered as an antipsychotic treatment. As reviewed Ahmed et al. the evidence remains inconclusive despite promising results. Two clinical trials have produced positive outcomes, while another trial failed to do so.

Compared to the cannabis-psychosis association, the link between cannabis and depression is less firmly established. In their review paper, Langlois et al. observed a bidirectional relationship between cannabis use and depression; although most studies showed an association, the link was not always observed. The risk for depression is possibly higher in people with heavy use of cannabis and those having initiated their consumption in early adolescence. While cannabis use is associated with a worsened prognosis in individuals with major depressive disorder (MDD), the link to suicide remains controversial. Data are insufficient in some areas, including with respect to the psychological treatment of CUD in MDD patients, the antidepressant potential of CBD, and mechanisms underlying the cannabis-depression association. Regarding the latter issue, Blum et al. argue that this association is due to the development of cannabis-induced hypodopaminergic anhedonia, as evidenced by positron emission tomography studies. If cannabis use increases the risk of experiencing anxio-depressive symptoms, one might expect cannabis abstinence to be associated with improvements in these symptoms. To investigate this possibility, Cooke et al. performed a study in non-treatment seeking adolescents who were randomized to 4 weeks of abstinence (achieved through contingency management) or ongoing consumption. Both groups had lower levels of anxiety and depression at thprovide doi linke study endpoint, and there were no between-group differences. Among the several reasons that could explain these results, the authors note that the recruited sample was composed of people with recreational use of cannabis. The recruitment of CUD individuals may have produced different results. Finally, Dellazizzo et al. reviewed evidence regarding the potential link between cannabis use and violence. Their meta-analyses demonstrated that cannabis is a potential risk factor for violent behaviors in youths and in people with psychotic disorders. The limitations of the studies performed in the field are discussed, most particularly in the case of studies performed in individuals with psychotic disorders (e.g., cross-sectional studies failing to properly control for potentially confounding factors). Two main explanatory models are presented: a pharmacological model whereby violence results from the pharmacological effects of cannabis; and a social model, whereby violent behaviors are the result of the social habits associated with the use of an illegal substance.

Cannabis may impair cognition, which may in turn impact academic and work achievement, and increase the risk for car accidents. Bourque and Potvin summarize the evidence on both the acute and residual effects of cannabis on cognition. Based on a previous meta-analysis (5), they show that acute intoxication with cannabis/THC is associated with prominent impairments in verbal memory and working memory. Impairments in speed of processing and executive functioning have also been observed across studies. Regarding potential residual effects of cannabis on cognition, deficits are typically mild to moderate, and most probably reversible. These conclusions may be misleading, however, considering that cross-sectional studies on cannabis have mostly focused on use rather than CUD. High-quality longitudinal studies have shown that cannabis use is mostly associated with deficits in verbal learning and executive functioning. The effects of cannabis on cognition have led investigators to identify the neural mechanisms underlying harmful effects. As reviewed by Morie and Potenza functional magnetic resonance imaging studies on executive functions demonstrate that cannabis use is associated with alterations in activity in frontal and cingulate regions; however, results are heterogeneous, and it remains to be determined if alterations are primary or secondary to cannabis use. Compared to *recreational* cannabis use, much less is known about the cognitive effects of cannabis use for *medical* purposes. To address this issue, Eadie et al. performed a scoping review of trials involving patients with neuropathic pain who were treated with smoked, vaporized or sublingual THC. The evidence indicated a cognitive decline among THC patients, mostly in a dose-dependent manner. However, the cognitive differences between THC and placebo groups were no longer different after 4 h of recovery. In theory, several factors may influence this general trend, including THC dose, the route of THC administration, interactions of THC with other drugs, CBD content and tolerance to THC, genetic factors and comorbidities. Their respective roles will need to be determined in future studies examining the cognitive effects of medical cannabinoids.

Among its acute effects, cannabis/THC impairs driving-relevant cognitive functions, including distance estimation, reaction time, vigilance, and processing speed. Likewise, most experimental studies reviewed Pearlson et al. show that acute cannabis/THC intoxication significantly impairs driving abilities, as measured in the laboratory. Meta-analyses have also shown that acute cannabis consumption increases the likelihood of motor vehicle accidents. The risk is not as elevated as in the case of alcohol; however, the combination of cannabis and alcohol seems to be particularly harmful. Increased frequencies of driving under the influence have been reported in some jurisdictions having legalized cannabis. As individuals consume cannabis products with higher potencies, it is reasonable to expect that more cannabis-related motor vehicle crashes will occur. The association between cannabis use and motor vehicle accidents is a major public health concern, since no reliable detection method of cannabis intoxication is available. THC is highly lipophilic, and as a result, serum or plasma THC levels do not predict well performance impairment. Current initiatives on new cannabis detection methods are discussed. Notwithstanding the growing diversification of cannabis forms and their routes of administration, the impact of these cannabis products on driving abilities has been understudied. This is the case, among others, of THC concentrates (e.g., dab, wax, shatter) which usually contain very high levels of THC. In an uncontrolled experimental study involving 65 individuals experienced in the use of concentrates, Hitchcock et al. sought to investigate this question. Using a mobile laboratory to measure motor abilities required for driving, participants were invited to use cannabis concentrates *ad-libitum*. Results showed that motor performance was impaired immediately after (e.g., arm speed and balance) and 1 h after (e.g., arm speed and leg speed) use of cannabis concentrates. These results highlight that cannabis concentrate use impairs driving-relevant motor abilities and raise significant issues regarding intoxication detection, particularly as THC plasma levels did not correlate with motor performance.

As observed by Matheson and Le Foll, there are scarce data on the harms of newer and/or more potent cannabis products, such as edibles, oils, concentrates, topicals and sprays. As legalization without restrictions may be as harmful to public health as prohibition, the authors propose to implement, in cannabis legalization models, (i) robust data collection to monitor harms associated with new cannabis products; (ii) early restrictions on cannabis edibles and high-potency products until safety data are gathered; and (iii) proper labeling of these cannabis products to clearly communicate dose information and health risks. As voiced by Crocker et al., another area requiring further research relates to the risk of emergency department (ED) visits. Although preliminary, an increase in cannabis-related ED visits has been described in Colorado, Nevada and Canada after cannabis legalization. Mental adverse events precipitating ED presentations include anxiety, agitation, suicidal thoughts and psychotic symptoms.

Together, the articles in this topic cover a broad range of considerations relating to the legalization of cannabis for recreational and medical purposes. As multiple jurisdictions progress with such legalization, appropriate support for research, prevention, treatment and policy initiatives should be made available to promote the public health.

## Author Contributions

SP wrote the manuscript. YK, AB, and MP provided critical comments. All authors approved the final version of the manuscript.

## Conflict of Interest

SP is holder of the Eli Lilly Canada Chair on Schizophrenia Research. The remaining authors declare that the research was conducted in the absence of any commercial or financial relationships that could be construed as a potential conflict of interest.

## Publisher's Note

All claims expressed in this article are solely those of the authors and do not necessarily represent those of their affiliated organizations, or those of the publisher, the editors and the reviewers. Any product that may be evaluated in this article, or claim that may be made by its manufacturer, is not guaranteed or endorsed by the publisher.
